# Drug perturbation gene set enrichment analysis (dpGSEA): a new transcriptomic drug screening approach

**DOI:** 10.1186/s12859-020-03929-0

**Published:** 2021-01-12

**Authors:** Mike Fang, Brian Richardson, Cheryl M. Cameron, Jean-Eudes Dazard, Mark J. Cameron

**Affiliations:** 1 Department of Population and Quantitative Health Sciences, Case Western Reserve University School of Medicine, Wolstein Research Building, 2103 Cornell Road, Suite 1-314, Cleveland, OH 44106-7295 USA; 2 Department of Nutrition, Case Western Reserve University, Cleveland, OH USA; 3 Center for Proteomics and Bioinformatics, Case Western Reserve University, Cleveland, OH USA; 4 Systems Biology and Bioinformatics Program, Case Western Reserve University, Cleveland, OH USA

**Keywords:** Transcriptomics, Gene set enrichment analysis, Drug discovery

## Abstract

**Background:**

In this study, we demonstrate that our modified Gene Set Enrichment Analysis (GSEA) method, drug perturbation GSEA (dpGSEA), can detect phenotypically relevant drug targets through a unique transcriptomic enrichment that emphasizes biological directionality of drug-derived gene sets.

**Results:**

We detail our dpGSEA method and show its effectiveness in detecting specific perturbation of drugs in independent public datasets by confirming fluvastatin, paclitaxel, and rosiglitazone perturbation in gastroenteropancreatic neuroendocrine tumor cells. In drug discovery experiments, we found that dpGSEA was able to detect phenotypically relevant drug targets in previously published differentially expressed genes of CD4+T regulatory cells from immune responders and non-responders to antiviral therapy in HIV-infected individuals, such as those involved with virion replication, cell cycle dysfunction, and mitochondrial dysfunction. dpGSEA is publicly available at https://github.com/sxf296/drug_targeting.

**Conclusions:**

dpGSEA is an approach that uniquely enriches on drug-defined gene sets while considering directionality of gene modulation. We recommend dpGSEA as an exploratory tool to screen for possible drug targeting molecules.

## Background

Drug discovery and/or screening can be an expensive and time-consuming endeavor with traditional methods relying on testing in in vitro or in vivo models or via screening of organs and tissues using synthetic molecules [1]. In some cases, the expense of a traditional drug development approach may overwhelm the resources available and make cost–benefit discussions challenging when bringing a new therapeutic to market [2]. Therefore, developing cost-efficient in silico strategies to screen for drug candidates that may be efficacious in treating human disease may result in novel or repurpose-able therapies [3]. With the rise of integrated -omics technologies, phenotypic screening [4], network-based [5], and literature mining [6, 7], new approaches that take advantage of large data-driven methodologies are at the forefront of drug screening [2]. Taking advantage of available knowledge, we propose a transcriptomically-driven drug screening approach that utilizes enrichment methods to determine candidate therapeutics.

Discovery-based enrichment methods can be used for finding matching transcriptomic signatures of drug-disease comparisons [8]. One approach, referred to as the signature reversion principle, has been successful in diverse therapeutic settings [9–11]. It assumes that a drug induced gene expression signature will be correlated with change of the transcriptome in disease to a healthy or healthier state [2]. Our premise is that a negatively correlated gene profile of a drug-perturbated transcriptome can be exploited in in silico drug screening methodologies.

Gene set enrichment techniques are well established in providing biological context in -omics studies, particularly in transcriptomic studies where summarizing the overall biology of a particular contrast or linear model by pathways enhances interpretability. We have used gene set enrichment techniques in a variety of transcriptional studies that compare or contrast the human host response to infectious or chronic illness [12–14]. Of the various enrichment approaches [15], Gene Set Enrichment Analysis (GSEA), Database for Annotation, Visualization and Integrated Discovery (DAVID) and Gene Ontology (GO) are gold standards in pathway and gene set enrichment for transcriptomic analyses [16–19], but unfortunately, their direct application in drug screening may not be ideal due to the lack of incorporation of drug-gene modulatory information. While other popular approaches such as gene2drug, DSEA, sscMAP, L1000cds, and CMAP-native methods may include such information, they lack the statistical rigor of GSEA [20–24]: none perform error rate analysis, calculate score normalizations, provide enrichment driver genes, or are tailored for transcriptomic analyses.

By performing enrichment on disease-associated gene signatures while using drug perturbation defined gene sets, entire transcriptomes can be probed for potential drugs or therapeutics. We propose a modified version of GSEA, namely drug perturbation GSEA (dpGSEA), to perform a unique drug-defined gene set enrichment analysis for screening therapeutics downstream of transcriptomic or proteomic studies. We describe dpGSEA as an analysis tool that emphasizes enrichment of counteracting gene expression between drug-gene and disease-gene profiles and provides an easily interpretable set of statistics to determine effectiveness of screened drugs. By using proto-matrices to capture *a-*priori drug perturbated gene signatures rather than gene sets, we believe our approach is well suited for transcriptomic-based therapeutic screening and enrichment.

## Methods

**Fig. 1:**
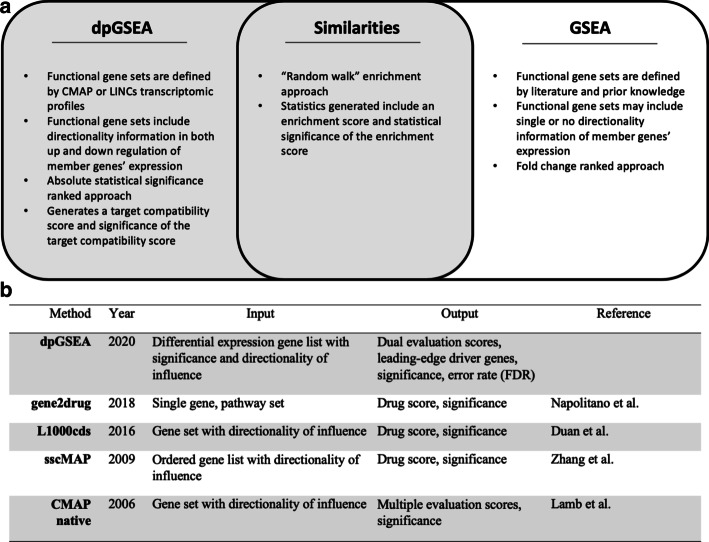
Comparisons between dpGSEA and similar approaches. **a** dpGSEA’s primary differences compared to GSEA include usage of a-priori gene set information derived from the Broad Institute’s connectivity map project (CMAP) and the library of integrated network-based cellular signatures (LINCS) projects organized as proto-matrices, an absolute statistical significance ranked approach rather than a fold change ranked approach, and a novel statistic to evaluate the drug target. Both approaches utilize a random walk running sum statistic to calculate enrichment scores. dpGSEA requires two inputs from the user to run. **b** dpGSEA is listed with comparable techniques that utilize GSEA-like approaches. Our approach uses the significance of a gene as well as directionality along with the generation of a novel statistic, the target compatibility score. We also show the driver genes for each drug

We provide a comparison between dpGSEA and related approaches in Fig. 1 and detailed definition, notation, framework, statistic, normalization and error rate notes in the Additional file 1: Methods.

### dpGSEA gene set priors

**Fig. 2:**
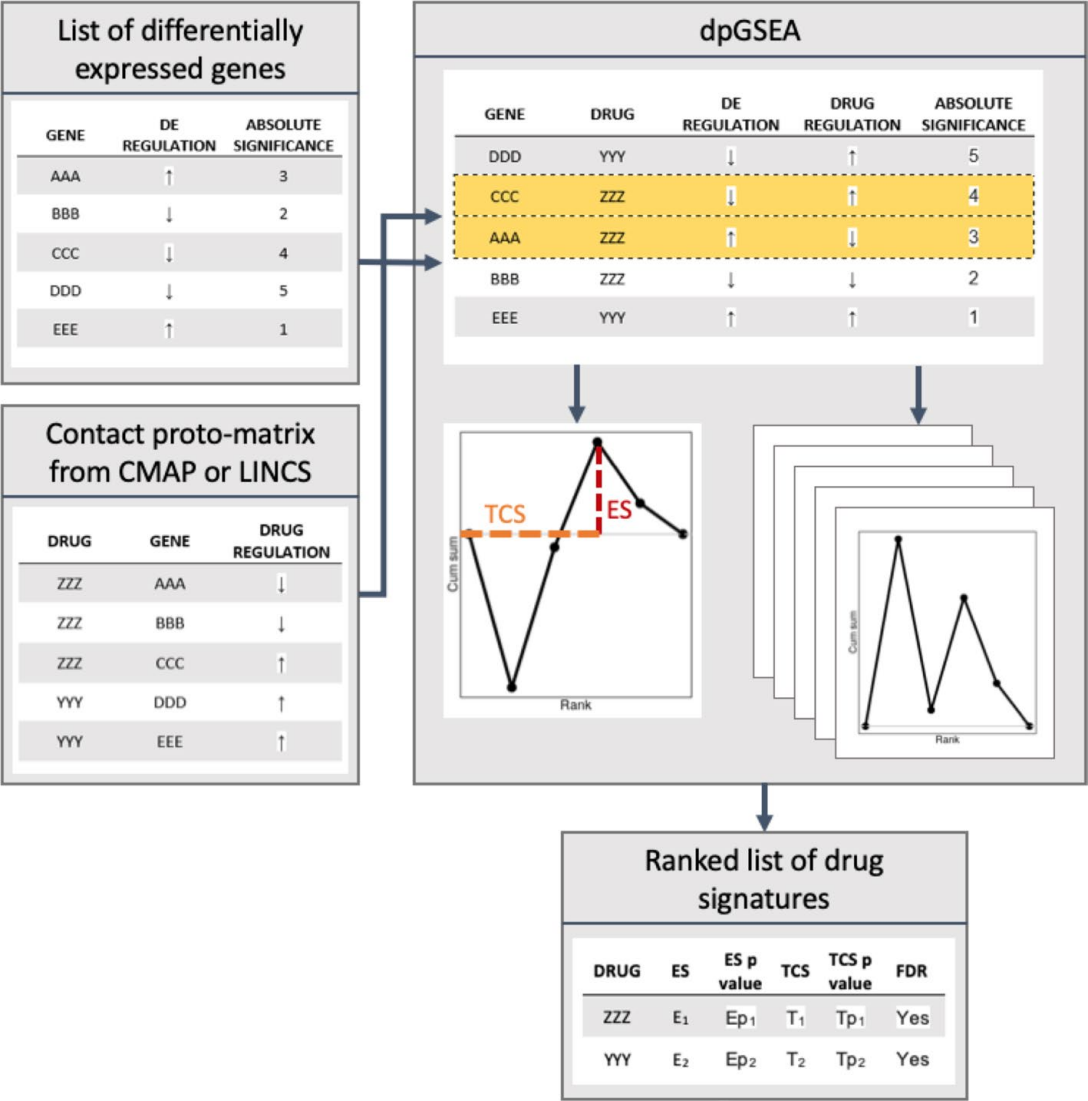
Overview of the dpGSEA pipeline and enrichment approach. Beginning from the left side of the diagram, the two primary inputs of dpGSEA are shown as tables. The top left table lists DEGs from, for example, a disease versus control study. The bottom left table contains the proto-matrix, which is analogous to MSigDB defined gene sets but contains a list of drug-gene actions rather than a gene set. dpGSEA merges the information in these tables by gene and ranks them by the absolute value of their significance. dpGSEA then estimates a running sum statistic based on drug-gene interaction and regulation. Highlighted in yellow are negatively correlated drug-gene interactions (opposing arrows). Enrichment distributions are formed [dotted red line, enrichment score (ES)] determining the maximum deviation of the running sum statistic plot, while the position of the maximum deviation (dotted orange line) represents the target compatibility score (TCS). dpGSEA then permutes the gene locations and generates new enrichment distributions along with null-enriched ES and TCS. The permutations are used to both normalize and generate statistical significance for each score. The output is a list of drugs ranked by their ES or TCS statistical significance (bottom center table). It should be noted that leading-edge genes are also included in the output (not shown)

An overview of the dpGSEA processing, including the proto-matrix is shown in Fig. 2. dpGSEA utilizes transcriptomic signatures of drug perturbated cell lines from Broad Institute’s connectivity map project (CMAP) and the library of integrated network-based cellular signatures (LINCS) projects to produce annotated gene sets rather than curated lists, like those from MSigDB [16, 24, 25]. These gene sets are organized into proto-matrices as defined by gene signature cutoffs of ranked top fold change or statistical significance. The proto-matrix itself contains information including genes acted on by a specific drug and the directionality in which it is influenced, that is, whether the drug induces up or down regulation of the gene.

To generate the proto-matrices, differential expression (DE) analysis using default LIMMA-voom parameters in Bioconductor was performed on the CMAP and LINCS data [26]. For each drug, a DE experiment was conducted using the correspondingly batched DMSO sample as controls, while remaining effects were linearly corrected. The resulting genes were ranked by fold change and statistical significance to generate a specific signature, that is, the top 10, 20 or 50 genes acted upon by a specific drug with the cell-line information retained (these are labeled as “Sig Rank 10” or “FC Rank 20”, etc. with the first label denoting fold change or significance and the last label denoting the number of top ranked genes).

### dpGSEA scoring statistics

Similar to the approach of GSEA (see Additional file 1: Methods), we consider a list *L* of annotated genes rank-ordered by increasing $T_{(j)}$, for $j \in \left\{ {1, \ldots ,p} \right\}$. Our method detects an enrichment of high values of $T_{(j)}$ in the positive tail of gene set $S_{k}$. This translates into finding evidence of a leading-edge subset in gene set $S_{k}$, in which the values of $T_{(j)}$ are maximal:

1. The traditional Enrichment Score, denoted $U_{k} = U(S_{k} ) = U\left( {T_{1} , \ldots ,T_{p} |S_{k} } \right) = ES_{k}$ which is calculated for each gene set $S_{k}$, as the maximum deviation from 0 of a weighted running sum, for $j \in \left\{ {1, \ldots ,p} \right\}$, in the gene set $S_{k}$, relative to its complement $\overline{S}_{k}$. Formally, our first gene-specific Global Test Statistic can be written as:
  1 $$ES_{k} = \mathop {\max }\limits_{{l \in \left\{ {1, \ldots ,p} \right\}}} \left| {v_{k} \left( l \right)} \right|,\;\;{\text{for}}\;\;l \in \left\{ {1, \ldots ,p} \right\}$$
  where $$v_{k} (l) = \frac{{\sum\nolimits_{j = 1}^{l} {\left| {T_{(j)} } \right|^{\omega } I\left[ {\sigma (j) \in S_{k} } \right]} }}{{\sum\nolimits_{j = 1}^{p} {\left| {T_{(j)} } \right|^{\omega } I\left[ {\sigma (j) \in S_{k} } \right]} }} - \frac{{\sum\nolimits_{j = 1}^{l} {I\left[ {\sigma (j) \notin S_{k} } \right]} }}{{p - \gamma_{k} }}$$.
  Where | | denotes the absolute value, $\max ( \cdot )$ denotes the maximum function with respect to gene index $l \in \left\{ {1, \ldots ,p} \right\}$, $\omega$ is a parameter describing the weight of the tail in the random walk (see remarks below), and $I\left[ {\sigma (j) \in S_{k} } \right]$ is the indicator function on whether the *j*th rank-ordered gene, belongs to gene set $S_{k}$ and is the inverse sign referring to the counter directionality for disease-gene and drug-gene, for $k \in \left\{ {1, \ldots ,K} \right\}$.
2. The Target Compatibility Score, denoted $U_{k} = U(S_{k} ) = U\left( {T_{1} , \ldots ,T_{p} |S_{k} } \right) = TCS_{k}$, which is calculated for each gene set $S_{k}$, for $k \in \left\{ {1, \ldots ,K} \right\}$, as the absolute distance between the point of maximum enrichment score and the point where the rank-ordered $T_{(j)}$ is minimal in absolute value, typically a zero fold-change or zero correlation gene index. This involves the computation of two gene indices: (1) the gene rank maximizer of the $ES_{k}$ statistic (leading edge upper bound), denoted $\hat{l}_{k}^{\max }$, and (2) the gene rank minimizer of the rank-ordered $T_{(j)}$, denoted $\hat{l}^{\min }$:
  2 $$TCS_{k} = \left| {\hat{l}_{k}^{\max } - \hat{l}^{\min } } \right|,$$

where $\hat{l}_{k}^{\max } = \mathop {\arg \max }\nolimits_{{l \in \left\{ {1, \ldots ,p} \right\}}} \left| {v_{k} (l)} \right|$ and $\hat{l}^{\min } = \mathop {\arg \min }\nolimits_{{j \in \left\{ {1, \ldots ,p} \right\}}} \left| {T_{(j)} } \right|$where $\arg \max ( \cdot )$ and $\arg \min ( \cdot )$ denote the maximizer and minimizer functions with respect to gene index $l \in \left\{ {1, \ldots ,p} \right\}$ and $j \in \left\{ {1, \ldots ,p} \right\}$, respectively.

### Normalization, significance, and error rate

Normalization places ES and TCS scores on respective comparable scales. A null distribution is created by gene label permutation of list *L* while retaining original gene label rank-ordering; this is performed for 1000 permutations. The normalization factor is the change of scale obtained by the mean of the scores generated by the permuted distributions, and the normalized score is then obtained by simply dividing the true score by this normalization factor. The significance of the true score is determined by the proportion of permuted scores that are greater than the true score, and our null hypothesis states that the true score is no different from those generated by random gene label permutation.

The multiple testing problem is addressed by our procedure carried out to control the False Discovery Rate (FDR). After a full experimental run of dpGSEA, the FDR is calculated by comparison of the proportion of all permuted null normalized scores for every drug screened greater than the specific score of a drug in question. This is performed for each ES and TCS respectively and is the approach utilized by GSEA.

### Testing dpGSEA

We approached testing dpGSEA in a two-fold manner. (1) We determined if dpGSEA was able to positively identify a perturbated drug from an external DE experiment through positively correlated gene modulation, as opposed to the signature reversion principle. (2) We used dpGSEA as intended, an exploratory tool for drug screening, to determine if the therapeutics detected have biological or phenotypic relevance to a disease in question.

For the first case, we tested third party gene signatures, not those from CMAP or LINCS, derived from gastroenteropancreatic neuroendocrine tumor cells (GEPNTs) perturbated by fluvastatin, parbendazole (against drug-defined gene sets present and generated from CMAP), paclitaxel, rosiglitazone (against drug-defined gene sets present and generated from LINCS), and doxorubicin (against drug-defined gene sets present and generated from both CMAP and LINCS) (Gene Expression Omnibus (GEO) #GSE98894) [27]. Drug perturbation DE for GEPNTs was performed using LIMMA-voom and matching signatures were detected using dpGSEA.

For the second case, drug screening, we applied dpGSEA to our recent study of differential gene expression in CD4+T regulatory cells (Tregs) from immune responders (IR) and nonresponders (INR) to antiviral therapy in HIV-infected individuals (GEO #GSE106792) [28]. This study assessed HIV-infected individuals for their ability to reconstitute the CD4+T cell pool in response to antiretroviral treatment and what candidate mechanisms were behind poor clinical outcomes and greater risk for morbidity and mortality with respect to INR status. Mitochondrial Treg mechanisms were implicated to be the cause of the cell cycle halting [28]. We analyzed this dataset with dpGSEA to determine whether we could identify drugs that may take advantage of differentially expressed genes (DEGs) involved in mitochondrial dysfunction or immune function as a whole in INRs.

## Results

Our case study results for detection of GEPNTs drug perturbations by dpGSEA that pass the FDR α = 0.05 threshold are shown in Table 1A and B for both ES and TCS, respectively, along with the specific proto-matrix used. It is worth mentioning that not every GEPNTs drug perturbation was positively identified by every proto-matrix by ES and TCS FDR thresholds, but we were able to positively identify all perturbations in most case with the exception of rosiglitazone by ES FDR and fluvastatin by TCS FDR. Paclitaxel perturbations were most frequently positively identified by both scores and primarily by significance-based LINCS proto-matrices, while other drugs varied in their positive findings with respect to the proto-matrix utilized.

**Table 1:** A rank-ordered list by (A) ES and (B) TCS *p* value of positively correlated validation tests using an external RNA-seq dataset

Drug	ES	NES	ES *p* value	Genes	Proto-Matrix
*A*					
paclitaxel_HT29	0.547	2.745	0.011	SCNN1A, AKR1C3	LINCS FC Rank 20
paclitaxel_HT29	0.742	2.863	0.014	CFAP70, C4BPB	LINCS Sig Rank 20
parbendazole_PC3	0.627	2.722	0.014	FSTL3, HIST1H2BG	CMAP FC Rank 20
fluvastatin_MCF7	0.295	2.509	0.016	IFIT1, MSMO1, INSIG1, HMGCR, IDI1, HSD17B7	CMAP FC Rank 50
fluvastatin_MCF7	0.377	2.515	0.016	SQLE, INSIG1, IDI1, SLCO4C1, MAP1S, RTEL1, PPIF, MAFK	CMAP Sig Rank 50
paclitaxel_MCF7	0.864	3.076	0.016	HSPB1	LINCS Sig Rank 10
paclitaxel_HT29	0.855	3.043	0.019	CFAP70, C4BPB	LINCS Sig Rank 10
doxorubicin_A375	0.529	2.588	0.020	MYB, CCL20, SLC27A2, MX2	LINCS FC Rank 20
paclitaxel_HELA	0.369	2.349	0.042	ABTB2, ZNF816, CASK	LINCS Sig Rank 50
paclitaxel_PC3	0.706	2.531	0.044	SIK1, GPM6A	LINCS FC Rank 20
parbendazole_MCF7	0.848	3.051	0.044	HIST1H2BG	CMAP Sig Rank 10

Drug	TCS	NTCS	TCS *p* value	Genes	Proto-Matrix
*B*					
rosiglitazone_HELA	0.999	1.362	0.010	INSIG1	LINCS Sig Rank 50
paclitaxel_MCF7	0.979	1.671	0.031	HSPB1	LINCS Sig Rank 10
parbendazole_MCF7	0.954	1.929	0.044	HIST1H2BG	CMAP Sig Rank 10
paclitaxel_HA1E	0.992	1.336	0.054	HIST1H2BD	LINCS Sig Rank 50
paclitaxel_MCF7	0.981	1.500	0.056	HSPB1	LINCS Sig Rank 20
paclitaxel_HT29	0.962	1.641	0.066	CFAP70, C4BPB	LINCS Sig Rank 10
paclitaxel_MCF7	0.982	1.370	0.088	HSPB1	LINCS Sig Rank 50
paclitaxel_HT29	0.964	1.474	0.101	CFAP70, C4BPB	LINCS Sig Rank 20
parbendazole_PC3	0.964	1.419	0.113	FSTL3, HIST1H2BG	CMAP FC Rank 20
paclitaxel_HELA	0.949	1.504	0.117	SIK1	LINCS FC Rank 20
paclitaxel_PC3	0.915	1.562	0.146	GAA	LINCS Sig Rank 10
doxorubicin_MCF7	0.849	1.717	0.150	S100A2	LINCS FC Rank 10

Each row represents a positively identified drug (rosiglitazone, fluvastatin, parbendazole, paclitaxel, or doxorubicin) by dpGSEA in GEPNTs perturbation versus GEPNTs DMSO control DE of our first test case. All findings shown pass an FDR threshold of α = 0.05 for ES in *A* and TCS in *B*. The leading-edge driver genes are listed in the “Genes” column and the specific proto-matrix the positive results were detected in are listed in the “Proto-Matrix” column. Positively identified paclitaxel perturbations were most frequent while other drugs were found in only some of the proto-matrices utilized

Table 2 shows the most statistically significant ES drug discoveries for the INR versus IR case study where mitochondrial and immunological associated drugs were found. Oseltamivir-carboxylate, the active metabolite of Tamiflu, an antiviral, prevents the release of progeny influenza virions while simultaneously modulating human sialidases which have been found to be localized in the mitochondria and involved in the regulation of cell apoptosis [29, 30]. Ibutilide, an antiarrhythmic, has been shown to inhibit endoplasmic reticulum and mitochondrial stress mechanisms [31]. These findings are consistent with the INR mitochondrial dysfunction while showing targetable transcription that may increase antiviral activity and/or prevent cell cycle disruption of Treg function. Notably, other drugs within statistical significance of 0.05, such as fibronil and telmesteine (p = 0.015, p = 0.017 respectively) have targets (CPT1A, and IDH2 respectively) suggested to be representative of fatty acid oxidation and energy production of mitochondrial dysfunction congruent with previous research [28].

**Table 2:** A rank-ordered list of the most statistically significant ES for drugs found when performing an enrichment for INR vs. IR Treg cells using top 50 *p* value rank proto-matrix (derived from LINCs)

Drug	NES	ES *p* value	NTCS	TCS p value	Gene
bendamustine_HT29	3.13	< 0.001	0.88	0.359	TMEM106B, TMEM135, ELOVL6, KCNJ2, LSM6, TMEM126B, TGIF1, TXNDC9, MAPKAPK5
oseltamivir-carboxylate_MCF7	3.12	< 0.001	0.97	0.150	RDH11, ETS1, YAF2, UBE4B, SFPQ, FRYL
medrysone_PC3	3.00	< 0.001	0.92	0.267	FAM13B, BMPR1A, MYO7A, CAST, H2AFV, RABL6
luliconazole_HT29	3.42	< 0.001	0.90	0.314	RBM7, TMED7, NXT2, ATMIN, SUB1, NPTN, WASF2, CEP135, RDH14
doxofylline_MCF7	3.43	< 0.001	0.88	0.347	SUCLG2, TM9SF3, BRD7, RPL36, APPBP2, MRPL33, ARL5A, TGFB3, LRRC15, HSP90B1, RPS25
ibutilide_HELA	3.23	0.001	0.91	0.294	EDEM3, RAD21, RAB35, ADGRF1, CBX1, SCN2B
cevimeline_HA1E	2.88	0.001	0.84	0.421	EDEM3, UBE4B, CTSD, LRRC15, ID2, SRSF11, TRIM21, NUCKS1, PARP1, SH2D3A
chrysin_HA1E	3.10	0.001	0.86	0.386	EDEM3, PBRM1, GANAB, MRPL33, LARP4, NKRF, BMI1, NOC3L, CORO2A
amtolmetin-guacil_MCF7	2.81	0.001	0.88	0.330	EDEM3, ASB13, SCAMP1, TRAPPC6A, EP300, MFSD6, AZIN1, CIAO1
triamterene_HT29	3.04	0.001	0.95	0.188	TMEM106B, UGDH, ELOVL5, BNIP1, ZBTB11, PIH1D1

Both normalized ES and normalized TCS along with their respective statistical significance are shown. The leading-edge genes are also displayed, indicating genes driving the enrichment scores. Note that the highest ranked result, oseltamivir-carboxylate, suppresses influenza virions production modulates human sialidases, known for mitochondrial involvement and regulation of cell apoptosis

**Fig. 3:**
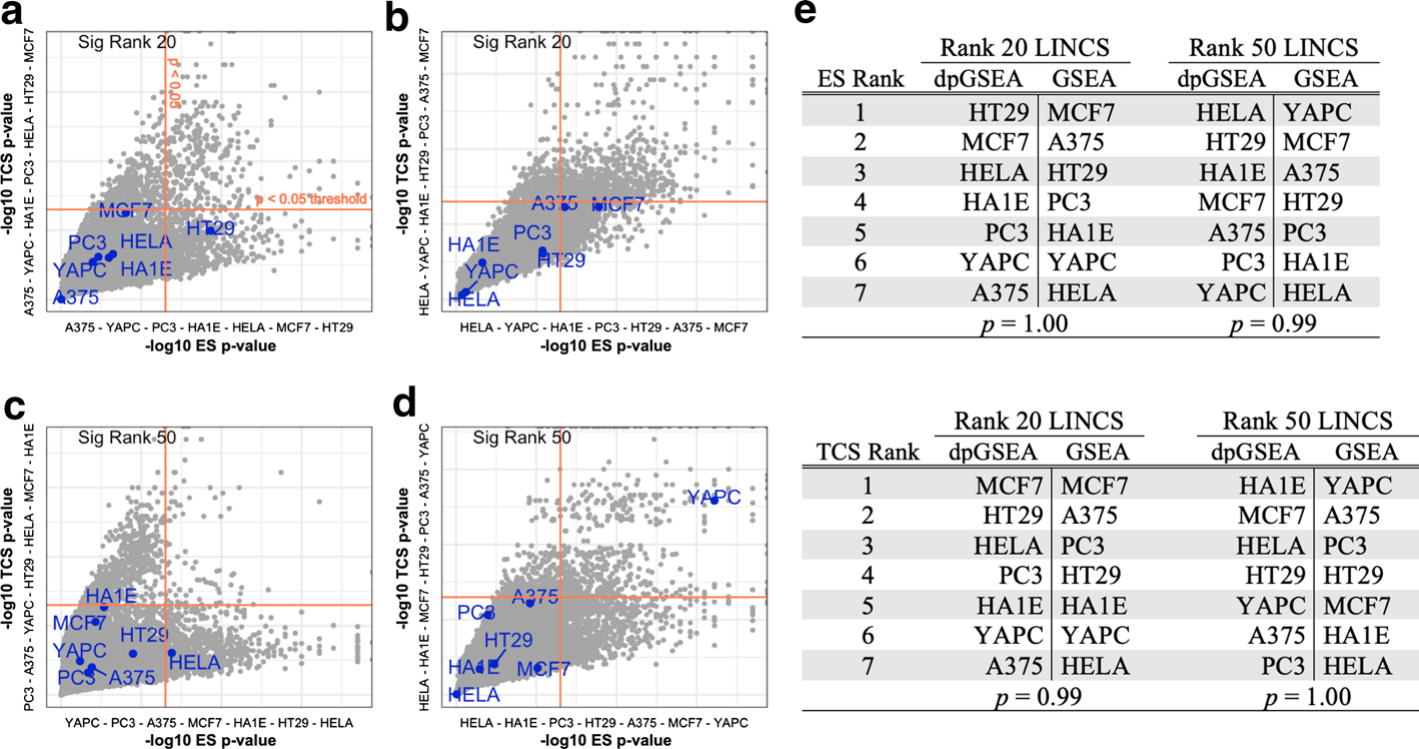
Comparisons between dpGSEA show statistically significant differences between enrichment results. Plots **a**–**d** show trends and comparisons between dpGSEA (**a**, **c**) and GSEA (**b**, **d**) for the top 20 and top 50 *p* value ranked proto-matrices (derived from LINCS data) identifying positively correlated genes. Plots **a** and **b** compare the top 20 ranked proto-matrices between dpGSEA and GSEA with each point representing an enriched drug in the final generated list. The labeled blue points all denote paclitaxel perturbated cell lines for the GEPNTs paclitaxel perturbation versus a GEPNTs DMSO control DE. The x-axis represents − log10 of the enrichment score (ES) *p* value and the y-axis − log10 of the target compatibility score (TCS) *p *value of corresponding perturbated drug cell line combination. The sub-axis lists the order of ascending significance for ES and TCS, respective of axis, that are also shown in *tables E*. The tables compare between the ranked orders for both ES and TCS with Wilcoxon signed rank test *p* values, suggesting the difference between dpGSEA and traditional GSEA results

When comparing dpGSEA with traditional GSEA, we find that the ranked ordering of paclitaxel perturbated cell lines are significantly different suggesting a substantial difference compared to our approach (Fig. 3). Wilcoxon signed rank tests are not significant (*p* > 0.90) when comparing dpGSEA with GSEA for both ES significance ranking and TCS significance ranking (Fig. 3e). Comparisons between ES and TCS rankings within dpGSEA and GSEA for comparable proto-matrices showed a maximum positional shift of 3 for perturbated cell line ranking (Top 50 Rank GSEA: MCF7 from 2nd to 5th) and most ranking shifts between ES and TCS within 2 spots (0 spot shift: 11; 1 shift: 9; 2shift: 5; 3shift: 1).

**Fig. 4:**
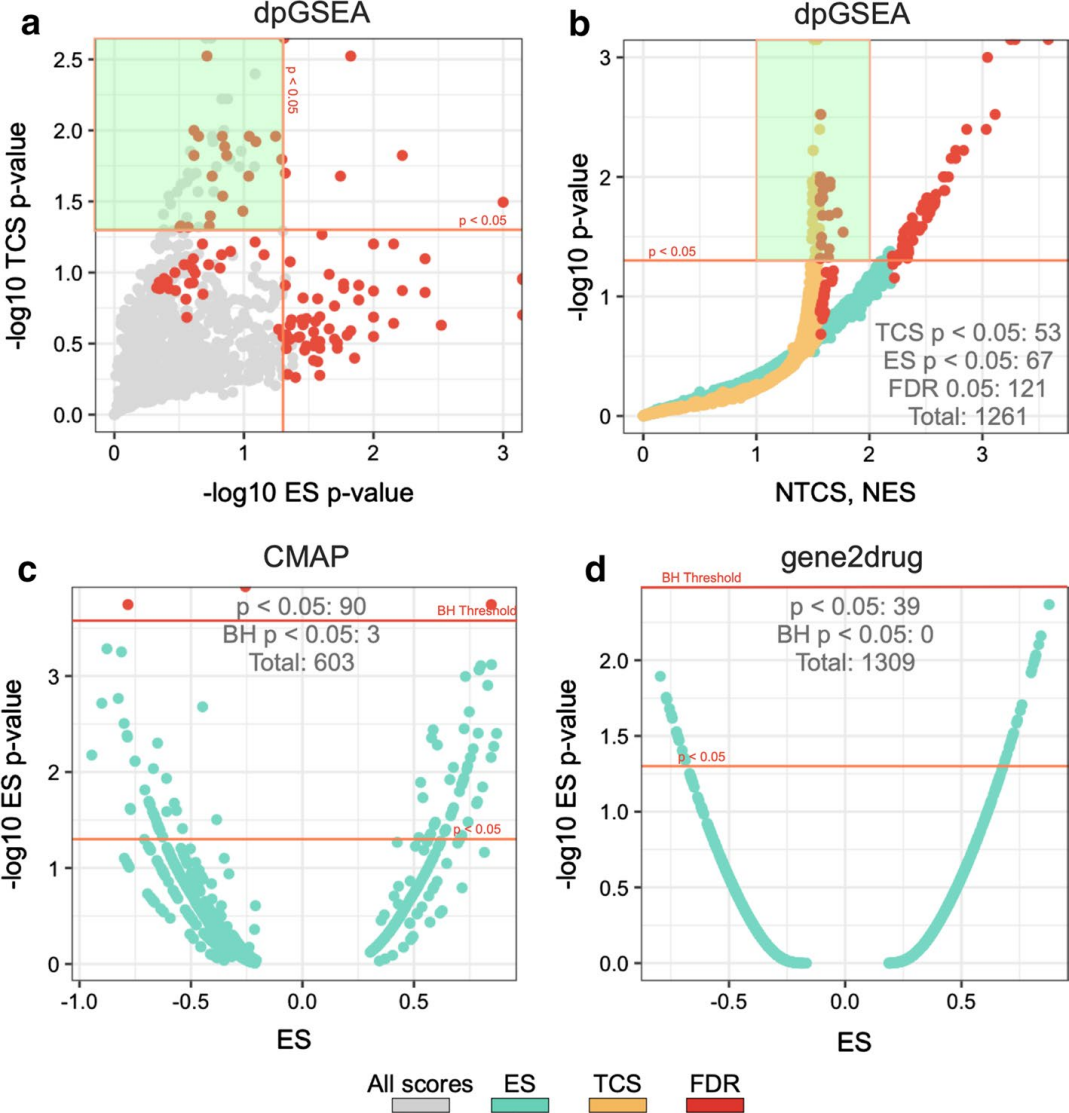
Comparisons between the trends of scores and significance for dpGSEA and the CMAP native and gene2drug approach. Each point within each plot represents a screened drug’s significance and score within an equivalent run for dpGSEA (plots **a**, **b**), CMAP native (plot **c**), and gene2drug (plot **d**). Screened drugs that pass a designated FDR significance threshold (0.05) are shown in red, and screened drugs highlighted in green show statistically significant findings unique to dpGSEA’s novel TCS. Total number of screened drugs within specific significance thresholds are also shown, and it should be noted that the number passing FDR α = 0.05 using the GSEA-defined FDR threshold (plot **a**, **b**) is 121 while those that pass the BH defined threshold are 3 and 0 for CMAP and gene2drug, respectively (plot **c**, **d**)

Figure 4 compares dpGSEA score and significance trends against those of the CMAP native and the gene2drug approaches. Two approaches were not included in our evaluation: the sscMAP approach is no longer available and the L1000cds approach does not provide a significance estimate. Here, we use the CMAP top 20 ranked significantly proto matrix as our *a-*priori signature for dpGSEA and correspondingly equivalent inputs (top 20 genes by significance) for other approaches. It should be noted that directionality is integrated into the dpGSEA enrichment approach producing only positive scores as reflected by the one-sided distribution of Fig. 4b. The ranked drugs that pass nominal GSEA-defined FDR, and Benjamini–Hochberg (BH)-defined FDR adjusted thresholds are also shown. Using the GSEA-FDR threshold [16], we find many drug screens that pass FDR = 0.05. This is in stark contrast to those approaches without inherent error rate analysis (Fig. 4c, d) that have few or no screened drugs that pass the BH-FDR threshold at the same level. Therefore, the dpGSEA screened drug results provide a richer and more reliable ranking of drugs for the clinician. In addition, it should be noted that this result is achieved by dpGSEA despite the fact that the GSEA-defined FDR procedure is inherently more conservative (less inductive of downward bias) than the BH-defined FDR procedures [32], especially in cases of lower α values (Additional file 2: Figure S1). Last but not the least, note the statistically significant findings of screened drugs (highlighted in green in Fig. 4) that pass a designated FDR significance threshold (0.05) unique to dpGSEA’s novel TCS statistic.

The distributions of scores and significance can be found in Additional file 3: Figure S2 and Additional file 4: Figure S3 which show trends between both normalized scores and their respective transformed *p* values along with leading edge gene set sizes. Each plot shows one completed run of dpGSEA with each drug and their respective scores and significance shown. We can see that, as expected, scores trend positively with significance, and that TCS significance tends to favor smaller sets of driver genes (R = 0.72) while ES does not (R = 0.03) in Additional file 4: Figures S3C and S3D. Furthermore, Additional file 4: Figure S3 shows a comparison between the positively identified fluvastatin perturbation for various proto-matrices. As *a-*priori signature sizes increase, we see fluvastatin migrate from lack of statistical significance to close and beyond TCS significance at *p* < 0.05 and ultimately to ES significance at *p* < 0.05. This may suggest that TCS is more capable of detecting enrichment for smaller gene set sizes.

## Discussion

The accurate portrayal of disease-gene and drug-gene complementary expression was the impetus for the development of dpGSEA. There are two features of dpGSEA that underscore its novelty in comparison to GSEA and other approaches, namely our indicator function denoting complementary disease-gene and drug-gene expression and the utilization of drug-derived gene set priors that include drug-gene modulation information. GSEA, in its current state, is not capable of producing results that can be interpreted with directionality of modulation by gene set priors for enrichment. Indeed, MSigDB gene sets, for example, only contain gene membership information. In cases where enrichment does take modulation of expression within a gene set into account, such as those defined in the C6 and C7 collections, the representation of a single biologically-defined gene set is dichotomized into up and down regulated groups [16]. This is less than ideal as interpretation of enrichment must be contextualized with two scores and two significance levels, making biological interpretations difficult in cases where two sets of estimates may not be congruent. Further, our results differ from those generated by traditional GSEA as shown by notable changes in rank of paclitaxel perturbated cell lines in Fig. 3, suggesting that the signature reversion principle plays a role when it comes to directionally influenced enrichment. In addition, with respect to GSEA but unlike other methods, we report FDR results in dpGSEA analyses that reflect a combination of both sensitivity and specificity metrics. However, in order to generate more specific accuracy metrics results like specificity and sensitivity, a simulation study of joint true drug perturbation and true DE (i.e. where the truth would be known for both) would be required to allow us to compare our candidate drug end results.

When compared to other drug screening approaches shown in Fig. 1b, we uniquely use both degree of modulation, as represented by DE significance, and directionality for enrichment. Methods such as gene2drug and DSEA require less conventional inputs, which will allow for application beyond transcriptomics but requires users to query with a single gene, a set of pathways, or a set of drugs without considering directionality of modulation [20, 21]. Although these approaches are versatile, dpGSEA takes advantage of the statistics generated in a DE experiment, making it uniquely postured for tackling transcriptomic drug screening. CMAP-native, L1000cds, and sscMAP approaches consider directionality, but not DE significance, and instead use ordered lists or sets [22–24]. Furthermore, we retain important aspects pivotal in GSEA’s success, such as score normalization and true FDR analysis in our approach [22, 24]. When comparing results between dpGSEA, CMAP native, and gene2drug, we see our approach provides for a greater number of drug screens that pass error correction. Our intrinsically less conservative GSEA-defined measurement of error is more appropriate for drug screening compared to the BH procedure. In our and other test cases the BH procedure shows strong bias towards exclusion of possible positive drug screens as shown in Fig. 4c, d, especially in cases of screens with high statistical significance, where the bias is most substantial (Additional file 1: Figure S1). The BH procedure, and others like it, is insufficient in understanding false discovery in these drug screening approaches which calls for an inherent method such as the one we have applied. Furthermore, for exploratory screenings, a less conservative error analysis that maintains strict statistical rigor is ideal. As a result, dpGSEA is fundamentally different from the aforementioned approaches, and we believe it can be an effective tool for drug screening for transcriptomic DE experiments. Furthermore, our novel statistic, TCS, serves as an alternative to the traditional ES by emphasizing gene rank with a DE experiment rather than statistical significance. It provides for another valid means of screening and, as shown in Fig. 4, elucidates a substantial number of otherwise ignored, but possibly important and effective, drug screens. This allows future studies another avenue for justification of exploration for a specific drug or gene target of interest if ES significance is not met.

When testing dpGSEA we were able to positively identify drug perturbations of paclitaxel, parbendazole, doxorubicin, rosiglitazone, and fluvastatin in GEPNTs, but we want to emphasize that dpGSEA’s primary purpose is discovery screening rather than identification. Our identification testing is a proof-of-concept for how our approach, in theory, can effectively apply the signature reversion principle in enrichment and detect drug perturbation signals for an external data set. We believe our true use-case test of dpGSEA on INR versus IR DE where mitochondrial and immunological associated drugs were found, is more revealing of dpGSEA approach’s capabilities.

Our scores, analogous to traditional GSEA scores, are rigorously generated while adjusted for false discovery to ensure the best possible accuracy. With respect to analytical studies based on DE analysis, i.e. all transcriptomic enrichment approaches, inferences made by dpGSEA will rely upon the validity of the prior DE results generated for the first stage of the dpGSEA framework. In line with this point, a recent study supports the importance of ranking statistics in GSEA. As the authors state, “An important parameter, which could affect the final result, is the choice of a metric for the ranking of genes. Applying a default ranking metric may lead to poor results*.*” [33] Hence, the important features of our approach include: (1) proto-matrices to capture more information, (2) a more accurate Local Test Statistic such as the Empirical Bayes Moderated Statistic estimated in implementations of Limma or edgeR packages, and (3) error rate control procedures such as FDR selection.

## Conclusions

We contend that our disease-gene and drug-gene complementary expression underpins the novel basis for dpGSEA, as well as the robust statistics controlled by multiple testing correction and the leading-edge driver genes generated by our approach. dpGSEA is an approach that uniquely enriches on drug-defined gene sets while considering directionality of gene modulation, and we recommend dpGSEA as an exploratory tool to screen for possible drug targeting molecules.

## Supplementary Information

**Additional file 1.** Expanded methods including statistical approach, overall framework, reiteration of LIMMA statistics, and FDR computation.

**Additional file 2: Figure S1.** A comparison between the critical values of GSEA-defined versus Benjamini–Hochberg defined error rate. Plots A, B, C, and D show analyses for the GSEA-defined FDR error rate in comparison with BH-defined FDR error rate for Fluvastatin using various proto matrices. Greatest departures of error rates (and critical values) are observed between GSEA-defined FDR and BH-defined FDR: at lower α (higher 1- α) levels, meaning that the GSEA-defined FDR error rate employed of our dpGSEA method tends to be less biased downward and therefore more conservative overall.

**Additional file 3: Figure S2.** ES and TCS significance trends for Fluvastatin screens are shown for GEPNTs for various proto matrices. The six plots show trends between ES statistical significance (x-axis) and TCS statistical significance (y-axis). In these case, 6 different proto-matrices derived from CMAP data identifying correlated signatures for fluvastatin in GEPNTs are shown with plots *B* and *E* reaching statistical significance at the 10% and 5% level for TCS, respectively. Plots *C and F* both reach statistical significance at the 5% level for ES with the orange line denoting statistical significance at the 5% level

**Additional file 4: Figure S3.** Trends normalized scores and leading-edge gene set size with their respective significance are shown for a single fun of dpGSEA. Four plots are shown representing the trends for one run of dpGSEA using a CMAP FC Rank 20 proto matrix. Plot *A* shows drug screens’ normalized ES (x-axis) and respective ES statistical significance (y-axis). Plot* B* shows the same but for normalized TCS scores. Plots* C* and *D* show the leading-edge gene sizes for both ES and TCS and their relationships with ES and TCS statistical significance suggesting that ES is robust with respect to leading-edge gene set sizes while TCS tends to favor smaller leading-edge gene set sizes

## Data Availability

Data are available in a GitHub repository, https://github.com/sxf296/drug_targeting.

## Abbreviations

BH: Benjamini–Hochberg
CMAP: Connectivity map project
DAVID: Database for Annotation, Visualization and Integrated Discovery
DE: Differential expression
DEGs: Differentially expressed genes
dpGSEA: Drug perturbation gene set enrichment analysis
ES: Enrichment score
FDR: False Discovery Rate
GEO: Gene expression omnibus
GEPNTs: Gastroenteropancreatic neuroendocrine tumor cells
GO: Gene Ontology
GSEA: Gene set enrichment analysis
IR: Immune responders
INR: Immune nonresponders
LINCS: Library of integrated network-based cellular signatures
Tregs: T regulatory cells
TCS: Target compatibility score
