# Supplementary material for: Drug perturbation gene set enrichment analysis (dpGSEA): a new transcriptomic drug screening approach
Source: BMC Bioinformatics. 2021 Jan 12;22:22. doi: 10.1186/s12859-020-03929-0 (PMC7805197; doi:10.1186/s12859-020-03929-0)
Supplement: Supplementary file 1 — Additional file 1. Expanded methods including statistical approach, overall framework, reiteration of LIMMA statistics, and FDR computation. [file 12859_2020_3929_MOESM1_ESM.docx]

**Supplemental Methods**

**Definitions and Notations.** dpGSEA, similar to traditional GSEA, uses inputs from three sources. Lowercase letters are used to denote realizations of random variables based on observed data, and uppercase letters to denote the corresponding random versions of these quantities):

- A real-valued $n\times p$ matrix $\mathbf{x}=\left(x_{ij}\right)_{n\times p}$ of expression values of *p* genes with *n* observations or measurements, either made on a continuous scale for array data or a discrete scale for sequencing data, and normalized, where $p\gg n$. We denote by $x_{ij}$ the expression of the *j-*th gene $\left(j\in\left\{1,\ldots,p\right\}\right)$ in *i-*th sample $\left(i\in\left\{1,\ldots,n\right\}\right)$, where the *p*-row vector $\mathbf{x}_{i}=\left(x_{i1}\ldots x_{ip}\right)^{T}$ denotes the *i-*th expression profile (sample), and the *n*-column vector $\mathbf{x}_{j}=\left(x_{1j}\ldots x_{nj}\right)^{T}$ the *j-*th gene expression profile (variable).
- A real-valued *n*-vector $\mathbf{y}=\left(y_{1}\ldots y_{n}\right)^{T}$ of univariate outcome response variable, where $y_{i}$ denotes the response in the *i-*th sample $\left(i\in\left\{1,\ldots,n\right\}\right)$, typically categorical (e.g. an experimental condition or group, or a class of phenotype of interest) or continuous (e.g. survival time).
- An *a-priori* defined collection of *K* Gene Sets $S=\left\{S_{k}\right\}_{k=1}^{K}$, $K>0$, where $S_{k}$ is the subset of column indices of $\mathbf{x}$ (annotated genes) such that $S_{k}\subseteq\left\{1,\ldots,p\right\}$, for $k\in\left\{1,\ldots,K\right\}$, defines a specific set of genes of interest. Formally, we define a Gene Set $S_{k}$ that is represented by a subset $S_{k}\subseteq\left\{1,\ldots,p\right\}$, for $k\in\left\{1,\ldots,K\right\}$, such that $j\in S_{k}$ if and only if the *j*-th gene is a member of $S_{k}$. The cardinal $\left|S_{k}\right|$ of Gene Set $S_{k}$ is denoted by $\gamma_{k}=\left|S_{k}\right|=\sum_{j=1}^{p} I(j\in S_{k})$. For any Gene Set $S_{k}$, its complement is denoted $\bar{S}_{k}$, and its cardinal by $p-\gamma_{k}=\left|\bar{S}_{k}\right|=\sum_{j=1}^{p} I(j\in\bar{S}_{k})$.

**Framework.** GSEA and other gene set enrichment methods have motivated the development of general statistical methodologies for large-scale inference for sets of variables[1]. We adopt the general framework of gene set enrichment[2] which applies to many more gene set enrichment methods including the original GSEA method. According to this framework, the goal is to detect an increased level of differentially expressed genes within a gene set $S_{k}$, for $k\in\left\{1,\ldots,K\right\}$, using a gene-specific test procedure that relies on a list of significant DEGs, to be identified by an *a-priori* gene-specific analysis. The whole process can be viewed as a two-stage procedure requiring the following two statistics, as well as a null distribution, and an error rate estimation procedure:

- A *Local Test Statistic* that measures the association between two variables: the expression profile of a gene $\mathbf{x}_{j}=\left(x_{1j}\ldots x_{nj}\right)^{T}$ for $j\in\left\{1,\ldots,p\right\}$ and the outcome response variable $\mathbf{y}=\left(y_{1}\ldots y_{n}\right)^{T}$. We denote the local statistic of the *j*-th gene by $T_{j}=T\left(\mathbf{x}_{j},\mathbf{y}\right)$ and its realization by $t_{j}$, for $j\in\left\{1,\ldots,p\right\}$. In a two-category experimental design, the local statistic can be for instance a *t*-statistic, an average fold change, or a sample correlation coefficient between $\mathbf{x}_{j}=\left(x_{1j}\ldots x_{nj}\right)^{T}$ and $\mathbf{y}=\left(y_{1}\ldots y_{n}\right)^{T}$.
- A *Global Test Statistic* that examines the local statistics of genes within a gene set $S_{k}$ in comparison to those in its complement $\bar{S}_{k}$, for $k\in\left\{1,\ldots,K\right\}$. We denote the global statistic by $U(S_{k})=U\left(T_{1},\ldots,T_{p}|S_{k}\right)$, abbreviated $U_{k}$, and its realization by $u(S_{k})$, abbreviated $u_{k}$*.*
- In addition, for each Global Statistic $U(S_{k})$ tested on a gene set $S_{k}$, for $k\in\left\{1,\ldots,K\right\}$, a hypothesis test is generated using a certain type (defined below) of *Null Hypothesis* on the Local Statistics $\left\{T_{j}\right\}_{j=1}^{p}$. In addition, due to the multiplicity of gene-specific hypothesis tests conducted on the *K* gene sets $S=\left\{S_{k}\right\}_{k=1}^{K}$, an *Error Rate* controlling procedure is applied to assess the final statistical significance of each individual gene set $S_{k}$, for $k\in\left\{1,\ldots,K\right\}$.

We consider a gene set in our dpGSEA analyses the drug-derived gene set $S_{k}$ of cardinal $\gamma_{k}$, and its corresponding drug candidate (denoted $D_{k}$), for $k\in\left\{1,\ldots,K\right\}$. Here, the outcome response variable $\mathbf{y}=\left(y_{1}\ldots y_{n}\right)^{T}$ is the presence of phenotype of interest, that is, a binary variable taking on two nominal [3] or categorical values [4].

**dpGSEA Statistics.** Our framework imposes that a null distribution is assumed or induced for the local statistics $\left\{T_{j}\right\}_{j=1}^{p}$. As mentioned above, no assumption is made in the following dpGSEA analyses about the dependence among the $\left\{T_{j}\right\}_{j=1}^{p}$. Formally, the null hypothesis of our Local Test Statistics states that all are identically distributed as follows:

$H_{0}:\text{ }T_{1},\ldots,T_{j},\ldots,T_{p}\sim^{id} F_{0}$ (1)

where $F_{0}$ corresponds to the “Class-I” gene-specific (gene-sampling) statistic[2], for which there is a lack of association between the expression profiles of all genes $\mathbf{x}_{j}=\left(x_{1j}\ldots x_{nj}\right)^{T}$ for $j\in\left\{1,\ldots,p\right\}$ and the outcome response variable $\mathbf{y}=\left(y_{1}\ldots y_{n}\right)^{T}$ of interest (or phenotype of interest). Here, our dpGSEA Local and Global Test Statistics reflect the enrichment found by matching the two sets of genes: the first one from a drug-derived expression profile list of genes (so called drug-derived gene set $S_{k}$ and corresponding drug candidate $D_{k}$), the second one from a ranked list of DEGs for the phenotype of interest (*L*).

**dpGSEA Local Test Statistic.** Our local test statistic employs derived moderated statistics[5, 6] in which posterior residual standard deviations are used in place of ordinary standard deviations to make inferences. The empirical Bayes approach is equivalent to shrinkage of the estimated sample variances towards a pooled estimate, resulting in far more stable inference when the sample size is small.

Consider a two groups experimental design with unpaired samples, unequal sample size, and unequal variance. Denote by *g* the *g*-th group, for $g\in\left\{1,2\right\}$, and let $y_{i}\in\left\{1,2\right\}$, for $i\in\left\{1,\ldots,n\right\}$, such that the value set of the outcome response variable $\mathbf{y}=\left(y_{1}\ldots y_{n}\right)^{T}$ is also $\left\{1,2\right\}$. Denote the *g*-th group sample index set by $C_{g}=\left\{i:y_{i}=g\right\}$, and the *g*-th group sample size by $n_{g}=\left|C_{g}\right|$, for $g\in\left\{1,2\right\}$. We use the Empirical Bayes moderated variance estimator of gene *j*, denoted $\tilde{s}_{j}^{2}$, for $j\in\left\{1,\ldots,p\right\}$, to describe the variability of the true gene-specific variances. Our Local Test Statistic can be written as a gene-specific *moderated t-test* as:

$T_{j}=\frac{\bar{X}_{1j}-\bar{X}_{2j}}{\tilde{s}_{j}\sqrt{\frac{1}{n_{1}}+\frac{1}{n_{2}}}}$ for $j\in\left\{1,\ldots,p\right\}$ (2)

Where $\tilde{s}_{j}=\sqrt{\frac{d_{0}s_{0}^{2}+d_{j}s_{j}^{2}}{d_{0}+d_{j}}}$, $d_{j}=n-p$ is the gene *j*-specific number of degrees of freedom, and parameters $d_{0}$, $s_{0}$ have to be estimated from the data (which is done automatically if using the implementation in Limma package), and where $s_{j}=\sqrt{\frac{\left(n_{1}-1\right)S_{1}^{2}+\left(n_{2}-1\right)S_{2}^{2}}{n_{1}+n_{2}-2}}$ is the ordinary gene *j*-specific unbiased estimator of the pooled standard deviation of the two groups, with the usual quantities: $\left(n_{1}-1\right)S_{1}^{2}=\sum_{i\in C_{1}} \left(x_{ij}-\bar{X}_{1j}\right)^{2}$, $\left(n_{2}-1\right)S_{2}^{2}=\sum_{i\in C_{2}} \left(x_{ij}-\bar{X}_{2j}\right)^{2}$, $\bar{X}_{1j}=\frac{1}{n_{1}}\sum_{i\in C_{1}} x_{ij}$, and $\bar{X}_{2j}=\frac{1}{n_{2}}\sum_{i\in C_{2}} x_{ij}$. The moderated local statistic $T_{j}$ is then used to conduct hypothesis testing against the null hypothesis $H_{0}:T_{j}=0$, and derive *p*-values, denoted $p_{j}$, for $j\in\left\{1,\ldots,p\right\}$.

**dpGSEA Global Test Statistic.** We consider the list *L* of column indices of $\mathbf{x}$ (annotated genes), rank-ordered by increasing Local Test Statistics $T_{(j)}$, for $j\in\left\{1,\ldots,p\right\}$, that is, by association between the expression profile of gene $\mathbf{x}_{j}=\left(x_{1j}\ldots x_{nj}\right)^{T}$, for $j\in\left\{1,\ldots,p\right\}$, and the outcome response variable $\mathbf{y}=\left(y_{1}\ldots y_{n}\right)^{T}$ of interest. $\left(T_{(j)}\right)_{j=1}^{p}$ denotes the rank-ordered tuple of Local Test Statistics, and by $\sigma:\left\{1,\ldots,p\right\}\to\left\{1,\ldots,p\right\}$ the rank-ordering bijection of them such that $\left(T_{j}\right)_{j=1}^{p}\mapsto^{\sigma} \left(T_{(j)}\right)_{j=1}^{p}$. dpGSEA uses two alternative Global Test Statistics that have the power to detect an enrichment of high values of Local Test Statistics in the tails of gene set $S_{k}$, relative to its complement $\bar{S}_{k}$. This translates into finding evidence of a leading-edge subset in gene set $S_{k}$, in which the values of Local Test Statistics are maximal:

1. The traditional Enrichment Score, denoted $U_{k}=U(S_{k})=U\left(T_{1},\ldots,T_{p}|S_{k}\right)=ES_{k}$ which is calculated for each gene set $S_{k}$, for $k\in\left\{1,\ldots,K\right\}$ as the maximum deviation from 0 of a weighted running sum (over all gene indices *j* of *L*) of the rank-ordered local test statistics $T_{(j)}$, for $j\in\left\{1,\ldots,p\right\}$, in the gene set $S_{k}$, relative to its complement $\bar{S}_{k}$. Formally, our first gene-specific Global Test Statistic can be written as:

$ES_{k}=\max_{l\in\left\{1,\ldots,p\right\}} \left|v_{k}\left(l\right)\right|$, for $l\in\left\{1,\ldots,p\right\}$ (3)

where $v_{k}(l)=\frac{\sum_{j=1}^{l} \left|T_{(j)}\right|^{\omega}I\left[\sigma(j)\in S_{k}\right]}{\sum_{j=1}^{p} \left|T_{(j)}\right|^{\omega}I\left[\sigma(j)\in S_{k}\right]}-\frac{\sum_{j=1}^{l} I\left[\sigma(j)\notin S_{k}\right]}{p-\gamma_{k}}$

where | | denotes the absolute value, $\max(\cdot)$ denotes the maximum function with respect to gene index $l\in\left\{1,\ldots,p\right\}$, $\omega$ is a parameter describing the weight of the tail in the random walk (see remarks below), and $I\left[\sigma(j)\in S_{k}\right]$ is the indicator function on whether the *j*-th rank-ordered gene, corresponding to the *j*-th rank-ordered local test statistic $T_{(j)}$, belongs to gene set $S_{k}$ or not, for $k\in\left\{1,\ldots,K\right\}$.

1. The Target Compatibility Score, denoted $U_{k}=U(S_{k})=U\left(T_{1},\ldots,T_{p}|S_{k}\right)=TCS_{k}$, which is calculated for each gene set $S_{k}$, for $k\in\left\{1,\ldots,K\right\}$, as the absolute distance between the point of maximum enrichment score and the point where the rank-ordered Local Test Statistic $T_{(j)}$ is minimal in absolute value, typically a zero fold-change or zero correlation gene index. This involves the computation of two gene indices: (i) the gene rank maximizer of the $ES_{k}$ statistic (leading edge upper bound), denoted $\hat{l}_{k}^{\max}$, and (ii) the gene rank minimizer of the rank-ordered Local Test Statistic $T_{(j)}$, denoted $\hat{l}^{\min}$. Formally, we can write our second Global Test Statistic TCS as:

$TCS_{k}=\left|\hat{l}_{k}^{\max}-\hat{l}^{\min}\right|$, (4)

where $\hat{l}_{k}^{\max}=\mathop{\mathrm{argmax}}\limits_{l\in\left\{1,\ldots,p\right\}} \left|v_{k}(l)\right|$ and $\hat{l}^{\min}=\mathop{\mathrm{argmin}}\limits_{j\in\left\{1,\ldots,p\right\}} \left|T_{(j)}\right|$

where $\mathrm{argmax}(\cdot)$ and $\mathrm{argmin}(\cdot)$ denote the maximizer and minimizer functions with respect to gene index $l\in\left\{1,\ldots,p\right\}$ and $j\in\left\{1,\ldots,p\right\}$, respectively.

**Normalizations.** This normalization effectively puts both Normalized Global Test Statistics (denoted $NU_{k}$, for $k\in\left\{1,\ldots,K\right\}$) on the same scale, and makes it possible to define a single null distribution (denoted $F_{0}^{NU}$) for the $NU_{k}$, for $k\in\left\{1,\ldots,K\right\}$. This assumes a null distribution (denoted $F_{0}^{U*}$) for the $U_{k}^{*}$, induced by gene permutation, where $F_{0}^{U*}$ corresponds to the aforementioned “Class-I” gene-specific (gene-sampling) null hypothesis[2], and $U_{k}^{*}$ denotes the Null Global Test Statistic. The empirical null distribution $F_{0}^{U*}$ is to be induced by generating all possible values of $U_{k}^{*}$ scores, denoted $U_{k}^{*b}$, for $b\in\left\{1,\ldots,B\right\}$, so that $F_{0}^{U*}=\left\{U_{k}^{*b}\right\}_{b=1}^{B}$, under a fixed permutation of genes, after reordering of the rank-ordered Local Test Statistics, denoted $\left(T_{(j)}^{*b}\right)_{j=1}^{p}$, and the row indices of $\mathbf{x}$ (annotated genes), rank-ordered accordingly in *L*. This is done for$B=1000$ permutations to have enough accuracy in the quantile estimates. The normalization factor is the change of scale obtained by dividing each Global Test Statistic $U_{k}$ by the expected value of $U_{k}^{*}$ under the null:

$NU_{k}=U_{k}/\text{E}\left(U_{k}^{*}\right)$ (5)

Similarly, the normalized dpGSEA Global Test Statistics are:

$NES_{k}=\frac{ES_{k}}{\text{E}\left(ES_{k}^{*}\right)}$, for $k\in\left\{1,\ldots,K\right\}$ (5)

$NTCS_{k}=\frac{TCS_{k}}{\text{E}\left(TCS_{k}^{*}\right)}$, for $k\in\left\{1,\ldots,K\right\}$ (5)

Statistical Significance, ***P*-values.** Under the hypothesis framework, a measure of significance for each Normalized Global Test Statistic $NU_{k}$, for $k\in\left\{1,\ldots,K\right\}$ is assessed by a *p*-value derived from a null distribution of a null hypothesis $H_{0}^{NU}$. Each Global Test Statistic is a measure of statistical evidence rejecting the null hypothesis that members of a given gene set $S_{k}$are randomly distributed in the rank-ordered list of annotated genes *L*. Formally, the null hypothesis $H_{0}^{NU}$ tests that all Normalized Global Test Statistics $NU_{k}$, for $k\in\left\{1,\ldots,K\right\}$, are identically distributed as the null distribution $F_{0}^{NU}$:

$H_{0}^{NU}:NU_{1},\ldots,NU_{k},\ldots,NU_{K}\sim^{id} F_{0}^{NU}$ (6)

Note that in multiple individual hypothesis testing, $H_{0,k}^{NU}:NU_{k}\sim F_{0}^{NU}$, there is a separate hypothesis and *p*-value for each Normalized Global Test Statistic *NU*, and each list $S_{k}$, for $k\in\left\{1,\ldots,K\right\}$, hence the notation $H_{0,k}^{NU}$, and $p_{k}^{NU}$, with dependency on *k* and *NU*.

Essentially, the *p*-value compares two statistics: one that is *observed*, and one that would have been observed under the null hypothesis, had the null hypothesis been true. Specifically, for each Normalized Global Test Statistic *NU*, and each list $S_{k}$, for $k\in\left\{1,\ldots,K\right\}$, the *p*-value is the probability

$p_{k}^{NU}=\Pr\left(NU_{k}^{*}>NU_{k}|H_{0,k}^{NU}=TRUE\right)$, (7)

where $NU_{k}$ is the observed Normalized Global Test Statistic, and $NU_{k}^{*}$ is the Normalized Global Test Statistic observed under the null hypothesis.

In our study, this translates into testing (alternatively) the null hypothesis $H_{0,k}^{NES}:NES_{k}\sim F_{0}^{NES}$ or $H_{0,k}^{NTCS}:NTCS_{k}\sim F_{0}^{NTCS}$ of whether each drug-derived gene set ($S_{k}$), for $k\in\left\{1,\ldots,K\right\}$, is randomly distributed within the rank-ordered list of DEGs (*L*) or not. Further, the corresponding dpGSEA *p*-values can formally be written as:

$p_{k}^{NES}=\Pr\left(NES_{k}^{*}\geq NES_{k}|H_{0,k}^{NES}=TRUE\right)$, for $k\in\left\{1,\ldots,K\right\}$ (7)

$p_{k}^{NTCS}=\Pr\left(NTCS_{k}^{*}\geq NTCS_{k}|H_{0,k}^{NTCS}=TRUE\right)$, for $k\in\left\{1,\ldots,K\right\}$ (7)

**Error Rates.** An Error Rate controlling procedure is applied to infer the final statistical significance of each individual drug candidate $D_{k}$ with its corresponding drug-derived gene set $S_{k}$, for $k\in\left\{1,\ldots,K\right\}$, while addressing the multiple (*K*) hypotheses testing problem. Specifically, an estimating procedure is carried out to control the False Discovery Rate (FDR), a critical measure of the False Positive Rate or Type_I Error Rate in a discovery list[7]. Here, dpGSEA provides a sorted list of drug candidates $D_{k}$ with their drug-derived gene set $S_{k}$, for $k\in\left\{1,\ldots,K\right\}$, denoted $R=\left\{S_{(k)}\right\}_{k=1}^{K}$, where $S_{(k)}$, for $k\in\left\{1,\ldots,K\right\}$, are the rank order gene sets obtained after sorting $\left\{S_{k}\right\}_{k=1}^{K}$ by increasing FDR.

The FDR can be calculated in two different ways: one can use the established procedure described in the original GSEA paper, further referred to as the GSEA-defined FDR and denoted *FDR_GSEA_*, or the less conservative traditional Benjamini-Hochberg procedure [8], further referred to as the BH-defined FDR an denoted *FDR_BH_* . Both FDR estimating procedures allow the assessment of significance of enrichment of a leading edge set of DEGs for the phenotype of interest in the given drug-derived gene set for hypothesis generation, as well as the prioritization of the drug-derived gene set versus other potential ones for drug discovery/repurposing.

Briefly, the *FDR_GSEA_* is calculated as follows. The same normalization factor is applied as above for each Global Test Statistic $U_{k}$ as well as for each Null Global Test Statistic $U_{k}^{*}$ by dividing each $U_{k}$ and $U_{k}^{*}$ by the expected value of $U_{k}^{*}$ under the null. This yields the normalized scores $NU_{k}=U_{k}/\text{E}\left(U_{k}^{*}\right)$, and $NU_{k}^{*}=U_{k}^{*}/\text{E}\left(U_{k}^{*}\right)$, respectively. Use this normalization scheme to get the empirical null distribution $F_{0}^{NU**}=\left\{\left\{NU_{k}^{*b}\right\}_{b=1}^{B}\right\}_{k=1}^{K}$. *FDR_GSEA_* is estimated as the number of falsely called drug-derived gene set $S_{k}$ (or falsely called drug candidate $D_{k}$), for $k\in\left\{1,\ldots,K\right\}$, divided by the number of drug-derived gene set $S_{k}$ (or drug candidate $D_{k}$) called significant. We compute the estimate of *FDR_GSEA_* *q*-value (denoted $q_{k}^{NU}$) of each drug-derived gene set $S_{k}$ (or drug candidate $D_{k}$), for $k\in\left\{1,\ldots,K\right\}$, for a given $NU_{k}^{*b(1-\alpha)}$ cutoff value corresponding to quantile $1-\alpha$ of $F_{0}^{NU**}$ as:

$\hat{q}_{k}^{NU}(\alpha)=\frac{\#\left\{NU_{k}^{*b}\geq NU_{k}^{*b(1-\alpha)}\right\}}{\#\left\{NU_{k}\geq NU_{k}^{*b(1-\alpha)}\right\}}$ (8)

**References**

1. Tamayo, P., et al., *The limitations of simple gene set enrichment analysis assuming gene independence.* Stat Methods Med Res, 2016. **25**(1): p. 472-87.

2. T Barry, W., A. B Nobel, and F. A Wright, *A statistical framework for testing functional categories in microarray data*. Vol. 2. 2008.

3. Colak, D., et al., *Integrated Left Ventricular Global Transcriptome and Proteome Profiling in Human End-Stage Dilated Cardiomyopathy.* PLoS One, 2016. **11**(10): p. e0162669.

4. Aandahl, E.M., et al., *Additive effects of IL-2 and protein kinase A type I antagonist on function of T cells from HIV-infected patients on HAART.* AIDS, 1999. **13**(17): p. F109-14.

5. Smyth, G.K., *Linear models and empirical bayes methods for assessing differential expression in microarray experiments.* Stat Appl Genet Mol Biol, 2004. **3**: p. Article3.

6. Phipson, B., et al., *Robust Hyperparameter Estimation Protects against Hypervariable Genes and Improves Power to Detect Differential Expression.* Ann Appl Stat, 2016. **10**(2): p. 946-963.

7. Benjamini, Y. and Y. Hochberg, *Controlling the false discovery rate: a practical and powerful approach to multiple testing.* J R Statist Soc, 1995. **57**(Series B): p. 289-300.

8. Storey, J.D., *A direct approach to false discovery rates.* J R Statist Soc, 2002. **64(3)**(Series B): p. 479–498.
