# Supplementary figures and images for: Drug perturbation gene set enrichment analysis (dpGSEA): a new transcriptomic drug screening approach
Source: BMC Bioinformatics. 2021 Jan 12;22:22. doi: 10.1186/s12859-020-03929-0 (PMC7805197; doi:10.1186/s12859-020-03929-0)

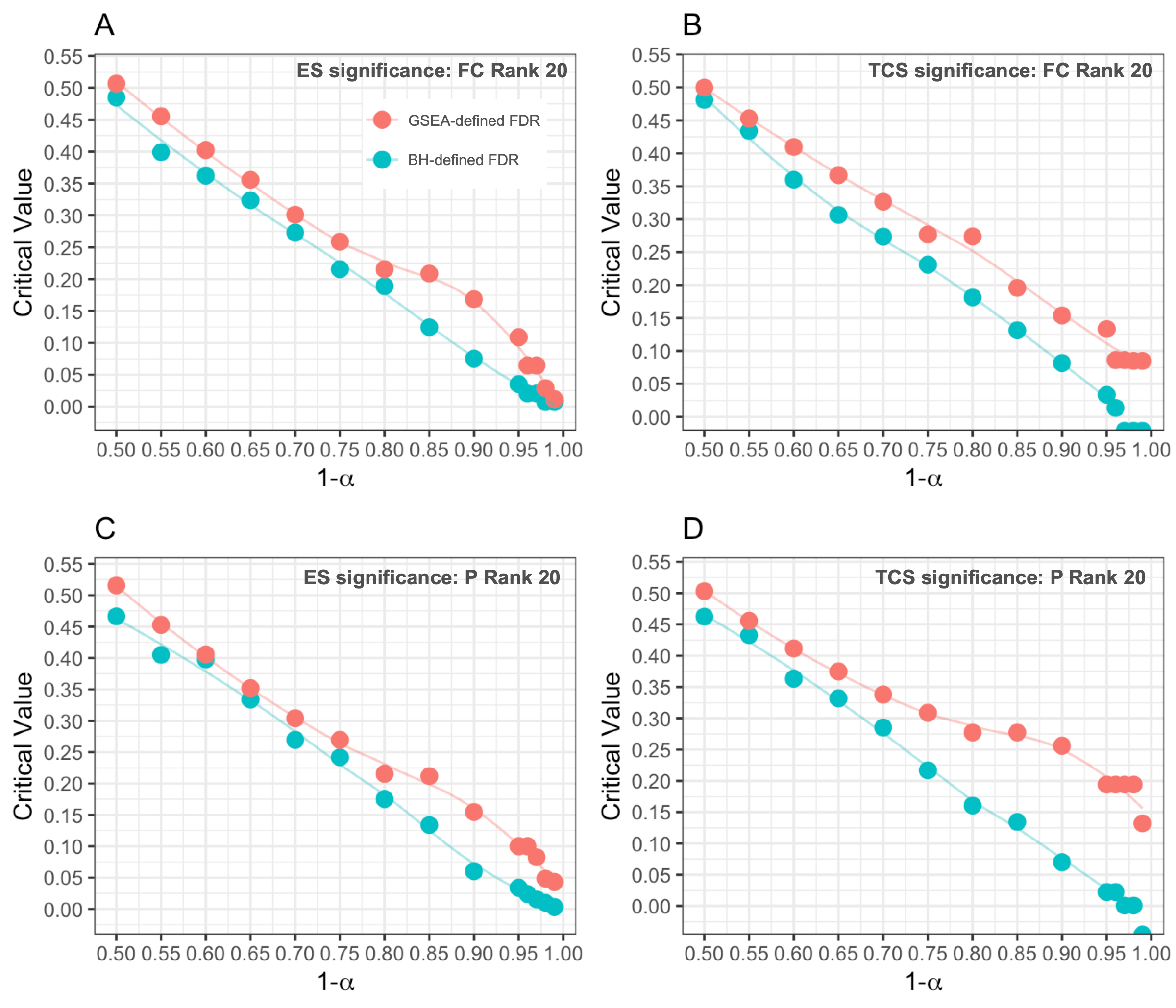

Supplement: Supplementary file 2 — Additional file 2: Figure S1. A comparison between the critical values of GSEA-defined versus Benjamini–Hochberg defined error rate. Plots A, B, C, and D show analyses for the GSEA-defined FDR error rate in comparison with BH-defined FDR error rate for Fluvastatin using various proto matrices. Greatest departures of error rates (and critical values) are observed between GSEA-defined FDR and BH-defined FDR: at lower α (higher 1- α) levels, meaning that the GSEA-defined FDR error rate employed of our dpGSEA method tends to be less biased downward and therefore more conservative overall. [file 12859_2020_3929_MOESM2_ESM.tiff]

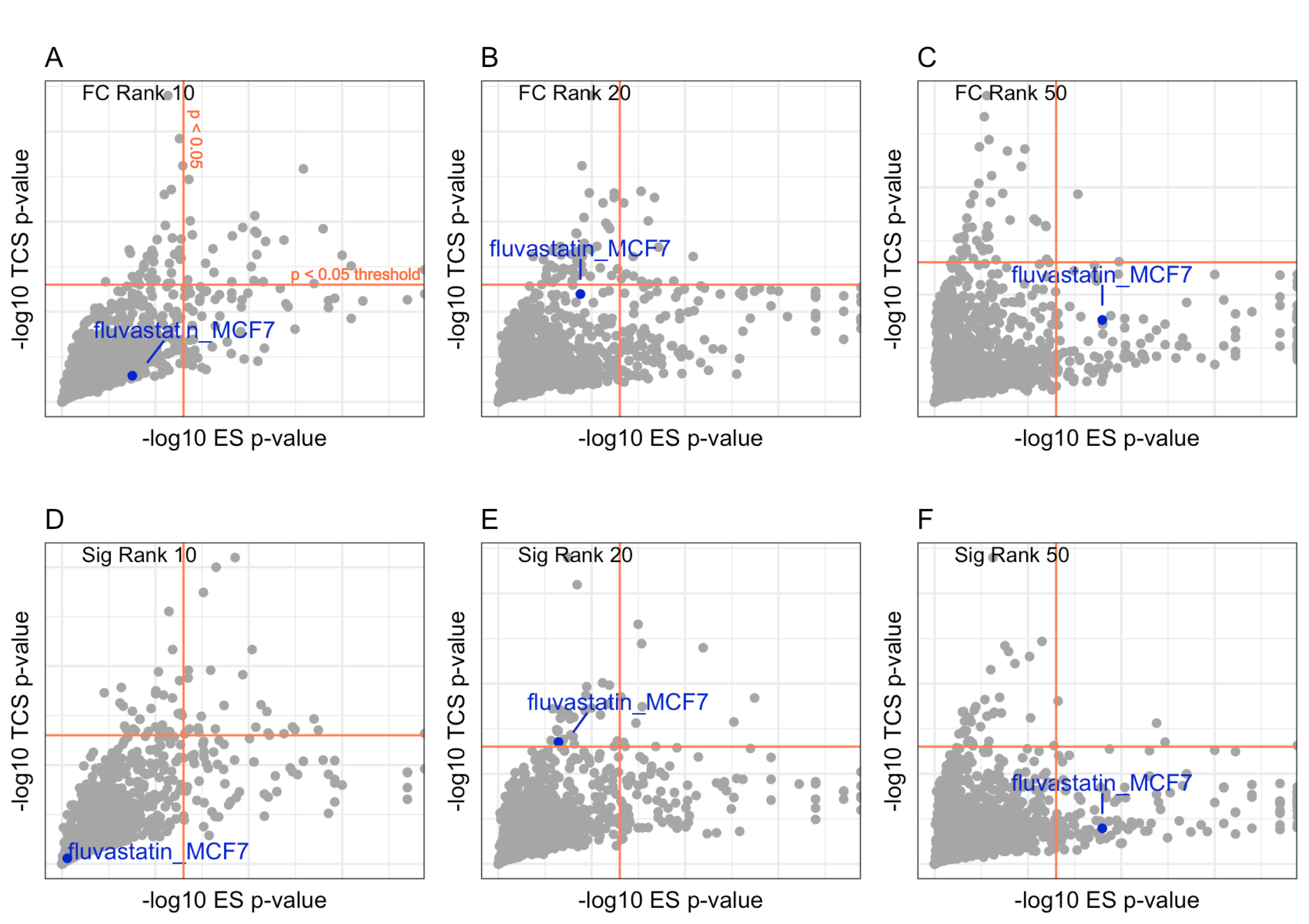

Supplement: Supplementary file 3 — Additional file 3: Figure S2. ES and TCS significance trends for Fluvastatin screens are shown for GEPNTs for various proto matrices. The six plots show trends between ES statistical significance (x-axis) and TCS statistical significance (y-axis). In these case, 6 different proto-matrices derived from CMAP data identifying correlated signatures for fluvastatin in GEPNTs are shown with plots B and E reaching statistical significance at the 10% and 5% level for TCS, respectively. Plots C and F both reach statistical significance at the 5% level for ES with the orange line denoting statistical significance at the 5% level [file 12859_2020_3929_MOESM3_ESM.tiff]

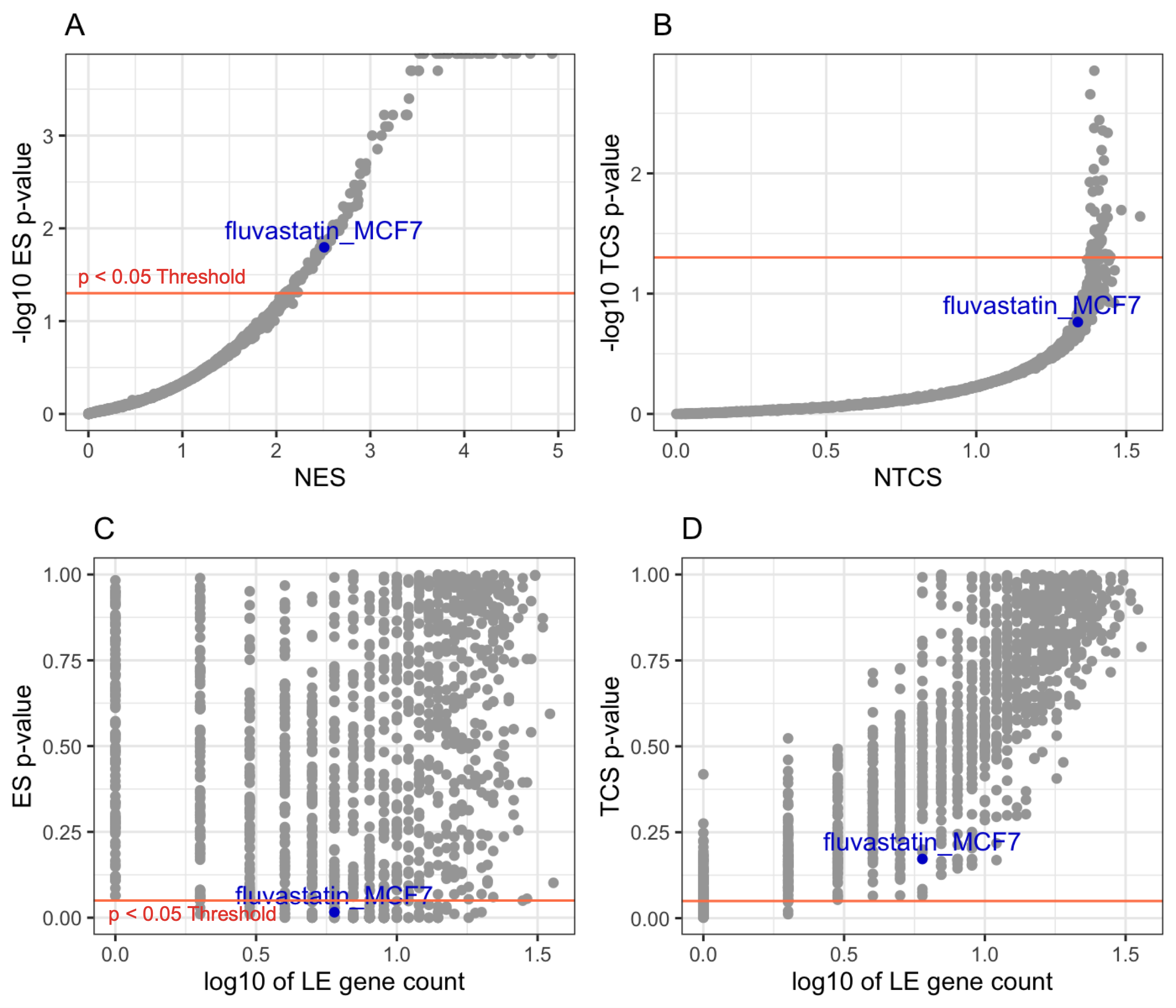

Supplement: Supplementary file 4 — Additional file 4: Figure S3. Trends normalized scores and leading-edge gene set size with their respective significance are shown for a single fun of dpGSEA. Four plots are shown representing the trends for one run of dpGSEA using a CMAP FC Rank 20 proto matrix. Plot A shows drug screens’ normalized ES (x-axis) and respective ES statistical significance (y-axis). Plot B shows the same but for normalized TCS scores. Plots C and D show the leading-edge gene sizes for both ES and TCS and their relationships with ES and TCS statistical significance suggesting that ES is robust with respect to leading-edge gene set sizes while TCS tends to favor smaller leading-edge gene set sizes [file 12859_2020_3929_MOESM4_ESM.tiff]
